# Manipulation of valve composition to elucidate the role of collagen in aortic valve calcification

**DOI:** 10.1186/1471-2261-14-29

**Published:** 2014-03-01

**Authors:** Karien J Rodriguez, Laura M Piechura, Ana M Porras, Kristyn S Masters

**Affiliations:** 1 Department of Biomedical Engineering, University of Wisconsin-Madison, 1550 Engineering Drive, #2152, Madison, WI 53706, USA

**Keywords:** Valvular interstitial cells, Calcific aortic valve disease, Collagen, Extracellular matrix

## Abstract

**Background:**

Extracellular matrix (ECM) disarray is found in calcific aortic valvular disease (CAVD), yet much remains to be learned about the role of individual ECM components in valvular interstitial cell (VIC) function and dysfunction. Previous clinical analyses have shown that calcification is associated with decreased collagen content, while previous *in vitro* work has suggested that the presence of collagen attenuates the responsiveness of VICs to pro-calcific stimuli. The current study uses whole leaflet cultures to examine the contributions of endogenous collagen in regulating the phenotype and calcification of VICs.

**Methods:**

A “top-down” approach was used to characterize changes in VIC phenotype in response to collagen alterations in the native 3D environment. Collagen-deficient leaflets were created via enzymatic treatment and cultured statically for six days *in vitro*. After culture, leaflets were harvested for analysis of DNA, proliferation, apoptosis, ECM composition, calcification, and gene/protein expression.

**Results:**

In general, disruption of collagen was associated with increased expression of disease markers by VICs in whole organ leaflet culture. Compared to intact control leaflets, collagen-deficient leaflets demonstrated increased VIC proliferation and apoptosis, increased expression of disease-related markers such as alpha-smooth muscle actin, alkaline phosphatase, and osteocalcin, and an increase in calcification as evidenced by positive von Kossa staining.

**Conclusions:**

These results indicate that disruption of the endogenous collagen structure in aortic valves is sufficient to stimulate pathological consequences in valve leaflet cultures, thereby highlighting the importance of collagen and the valve extracellular matrix in general in maintaining homeostasis of the valve phenotype.

## Background

Calcific aortic valve disease (CAVD) is characterized by severe thickening of the valve leaflets, extracellular matrix (ECM) disarray, and formation of calcified nodules deposited in the fibrosa, leading to valve stiffening and obstructed ventricular outflow [1]. Recent evidence suggests that this calcification is a cell-mediated, active process that involves elements of both dystrophic and ossific calcification, including expression of markers indicative of either myofibroblast or osteoblast differentiation, respectively [2,3]. There is mounting *in vitro* and *in vivo* evidence that the valve ECM may play a critical role in mediating these events [4-9]. Although several studies have highlighted the importance of the overall valve ECM structure in maintaining not only the mechanical, but also the biological, functions of the valve [6,7,9-11], the contribution of individual ECM components to CAVD etiology is not well understood.

Collagen is the major ECM component of the aortic valve, comprising approximately 50% of the total valve ECM composition by dry weight [12]. The most abundant types of collagen found in the aortic valve are type I (74%), type III (24%) and type V (2%) [13]. Collagen is mainly found in the fibrosa as interweaving, densely-packed bundles of fibers oriented circumferentially [14,15], where it serves to provide mechanical strength to the valve. The unique interweaving configuration of the collagen fibrils allows for load resistance and leaflet expansion during valve closure, and the maintenance of collagen homeostasis is therefore imperative for proper valve function.

In the native valve, collagen content decreases with age [16], and this event is accompanied by an increase in the prevalence of aortic valve calcification [17]. Among the many phenotypic changes that can occur during CAVD is the upregulated expression of matrix metalloproteinases 1, 3, 9 and 13 (MMP-1, MMP-3, MMP-9 and MMP-13) [18,19], all of which are associated with collagen degradation [20]. This ultimately results in extensive disorganization of the collagen fibers in the leaflet [21], thus compromising the structural integrity of the valve. Analysis of explanted calcified valves has shown that total collagen content in these valves is significantly lower than that found in age-matched normal valves [8]. These findings indicate that, during calcification, degradation of collagen exceeds its synthesis. Moreover, this increase in collagen degradation, specifically collagen type I, occurs in a localized manner around calcific nodules [8]. While this previous work suggests that collagen deficiency is associated with calcific events, it is unknown whether this phenomenon is sufficient to initiate further valve or VIC dysfunction.

These studies are complemented by *in vitro* work demonstrating that nodule formation by VICs is highly dependent upon ECM composition; specifically, some ECM components strongly support the formation of nodules in VIC cultures, while other ECM components, such as collagen, appear to inhibit nodule formation [7]. VICs cultured on collagen-coated surfaces have demonstrated resistance to calcification even after treatment with pro-calcific stimuli [7]. Together, these findings implicate VIC-collagen interactions as a crucial element in maintaining a healthy VIC phenotype, but much remains to be learned regarding the role of collagen in CAVD etiology.

Therefore, this work studies the importance of VIC-collagen interactions in the maintenance of a healthy VIC phenotype. We analyzed the VIC-collagen relationship via a “top-down” approach previously described by our lab [5] that allowed controlled manipulation of native ECM composition to characterize VIC phenotype in response to the introduction of a collagen deficiency in a native 3D environment. Understanding the individual contribution of collagen, a major valve ECM component, to valvular disease may not only help us better understand calcific valve etiology and identify potential targets for treatment, but also help to inform the design of scaffold materials for tissue-engineered valves.

## Methods

Unless otherwise stated, all products are from Sigma-Aldrich, St. Louis, MO. Data were compared using ANOVA with Tukey’s HSD post-hoc test, or a student’s t-test where appropriate, for which statistical significance was defined as P ≤ 0.05. Data are presented as mean ± standard deviation.

All tissues used in this study were acquired post-mortem from a commercial slaughterhouse, and were therefore not subject to institutional animal protocol approval. The slaughterhouse follows USDA and Humane Slaughter Act guidelines for care and slaughter of the swine.

### Depletion of collagen from native valve leaflets

The collagen content of native valve leaflets was altered via targeted enzymatic digestion, similar to previous work described for hyaluronic acid depletion [5]. Leaflets were excised from porcine aortic valves (Hormel, Inc., Austin, MN) and washed in a solution of Medium 199 (pH 7.4) supplemented with 200 U/ml penicillin and 200 μg/ml streptomycin. This was followed by a 1.5 hour incubation at 37°C with shaking in a solution of 96 U/mL collagenase type II (Worthington Biochemical Corp.), while control leaflets were incubated in wash solution for the same duration of time. Leaflets were rinsed numerous times in wash solution, denuded of endothelial cells, cut in half, and prepared for either Day 0 characterization or *in vitro* organ culture as described in subsequent sections. Untreated native leaflets were used as controls. Endothelial denudation was confirmed via detection of von Willebrand factor (rabbit anti-vWF, Dako, Carpinteria, CA) using standard immunohistochemical techniques.

### Validation of targeted collagen depletion method

To validate the effectiveness of collagenase treatment to selectively remove collagen from native valve leaflets, Day 0 enzyme-treated leaflets were frozen immediately following the collagenase treatment, freeze-dried for 72 hours, flash frozen in liquid nitrogen, pulverized, and incubated in a papain solution (19 U/mg papain (Worthington Biochemical), 10 mM cysteine in PBE buffer) overnight at 60°C with shaking. Following digestion, samples were homogenized by vortexing and used for detection of total collagen, hyaluronic acid (HA), sulfated glycosaminoglycans (sGAGs), and DNA. Total collagen was indirectly measured by detecting the total amount of hydroxyproline in the leaflet digest [22], wherein hydroxyproline accounts for 13.3% of total collagen [23]. The amount of sGAG was quantified by the dimethyl methylene blue (DMMB) assay and compared against chondroitin sulfate standard solutions as previously reported [24]. The amount of HA in the samples was determined using a HA quantitative test kit (Corgenix, Broomfield, CO). Total DNA content was measured by the Pico Green assay (Invitrogen, Carlsbad, CA). All results were normalized to leaflet dry weight. Swelling ratio of the leaflets was calculated as the leaflet wet weight divided by the leaflet dry weight. To conduct mechanical testing, enzyme-treated and control leaflets were cut into 4.5 mm wide longitudinal strips and mechanically tested in tension (Instron Model 5548 MicroTester) to calculate the leaflet Young’s modulus, as has been described elsewhere for the mechanical characterization of heart valves [25]. Testing was performed in an environmental chamber filled with PBS and held at 37°C. To visualize valve architecture following collagenase treatment, histological examination was performed on formalin-fixed leaflets that were embedded in paraffin and cut into 7 μm thick sections. Sections were stained with Movat’s pentachrome (Poly Scientific, Bay Shore, NY), and brightfield images were captured using an Olympus IX51 microscope.

### In vitro culture of whole leaflets

Untreated and collagenase-treated leaflets were prepared as described above and statically cultured attached to non-adhesive silicone bases at 37°C in VIC growth medium containing 15% FBS. Culture medium was changed every other day for a period of 6 days. After culture, leaflets were fixed or digested for analysis of cell proliferation, apoptosis, or phenotype via methods specified in subsequent sections.

### Proliferation and apoptosis

Proliferating VICs in cultured leaflets were visualized and quantified using the Click-It EdU Cell Proliferation kit (Invitrogen). EdU was added to the media on Day 5 of leaflet culture and incubated overnight. On Day 6, leaflets were fixed in 10% neutral buffered formalin for 1 hour, embedded in tissue freezing medium, flash frozen, and sectioned into 7 μm slices using a cryostat (HM 505-E, Microm International GmbH, Walldorf, Germany). Detection of EdU was achieved by a copper-catalyzed reaction resulting in fluorescence that was visualized using an Olympus IX51 microscope, and sections were counterstained with DAPI (1 μg/ml). The number of actively proliferating cells and total cells in leaflet sections was quantified using NIH ImageJ Software.

Apoptotic VICs in the leaflets were visualized using the TUNEL-based ApopTag Red In Situ Apoptosis Detection Kit (Millipore, Billerica, MA). After culture, leaflets were fixed in 10% neutral buffered formalin for 1 hour at RT followed by post-fixation in pre-cooled ethanol:acetic acid solution (2:1) for 5 minutes at -20°C. Tissue sections were prepared as specified above. After application of the equilibration buffer, TdT enzyme was incubated for 1 hour at 37°C. Subsequent TdT detection was performed by incubating with anti-digoxigenin conjugate for 30 minutes at RT. Apoptotic cells in DAPI-counterstained leaflet sections were visualized using an Olympus IX51 microscope. The number of apoptotic cells and total cells in leaflet sections was quantified using NIH ImageJ Software.

### Characterization of cell phenotype in leaflets

Histological analysis for markers of a diseased VIC phenotype was performed on collagen-depleted and control leaflets after six days of *in vitro* culture. Leaflets were fixed in 10% neutral buffered formalin and prepared for sectioning as described above. Tissue sections were washed four times with diH_2_O prior to immunohistochemical staining for alpha smooth muscle actin (α-SMA) or alkaline phosphatase (ALP). Sections were blocked overnight with a 5% BSA solution in PBS at RT, followed by application of primary antibodies diluted in 1% BSA in PBS: anti-α-SMA (mouse, clone 1A4, 5 μg/mL) and anti-ALP (mouse, clone AP-59, 1:1000). After overnight incubation at 4°C, the sections were rinsed in PBS and reacted with goat anti-mouse AlexaFluor 488 IgG conjugate (2 μg/mL; Invitrogen) for 1 hour. The leaflet orientation and region that is displayed is comparable across all photomicrographs.

Quantification of α-SMA and ALP was performed via western blotting. Tissue specimens were lysed in a 2% sodium dodecyl sulfate (SDS) lysis buffer, and the amount of total protein in the samples was quantified via the Micro BCA Assay (Pierce, Rockford, IL). Separation of proteins in the lysate was performed by gel electrophoresis using 4-12% Bis-Tris NuPage Gels (Invitrogen). Blotting onto a nitrocellulose membrane and subsequent blocking were performed using standard western blotting techniques. Blot membranes were then incubated with primary antibodies (anti-α-SMA, anti-ALP, and anti-β-Tubulin; rabbit, AbCam, Cambridge, MA) for 1.5 hours at RT with shaking and IRDye-conjugated secondary antibody (Li-Cor Biotechnology, Lincoln, NE) for 1 hour at RT with shaking. Infrared fluorescence was detected and quantified using the Odyssey Infrared Imaging System (Li-Cor).

### Quantification of calcific markers via qRT-PCR

Total RNA was isolated using the RNeasy Mini Kit (Qiagen, Valencia, CA). After six days of *in vitro* culture, leaflets were flash frozen in liquid nitrogen, pulverized and lysed as specified by the manufacturer. Construction of the cDNA was done by reverse transcribing 100 ng of the isolated RNA using the High Capacity cDNA Reverse Transcription Kit (Applied Biosystems, Carlsbad, California) according to manufacturer’s instructions.

Custom TaqMan Gene Expression assays for osteocalcin (OCN) and bone sialoprotein (BSP), later-stage markers of osteogenesis, were purchased from Applied Biosystems. For real-time PCR analysis, 1 μL of the cDNA was combined with 1 μL of TaqMan Gene Assay and TaqMan Master Mix, as specified by the manufacturer. The PCR cycling temperatures and times were also specified by the manufacturer (40 cycles of denaturing at 95°C for 15 seconds and annealing at 60°C for 1 minute). The PCR data were first normalized to GAPdh and then to the undepleted Day 6 leaflet control using the comparative C_T_ method (ΔΔ C_T_).

### Calcification staining

Tissue mineralization was detected by visualization of calcium deposition in the sections via von Kossa staining. Tissue sections were incubated in a 5% silver nitrate solution for 1 hour under ultraviolet light and counterstained with 0.1% nuclear fast red (Acros Organics) for 5 minutes. Calcium deposits were identified in photomicrographs by black staining. Calcium content was quantified by performing extraction of Alizarin Red S (ARS) staining [26]. In brief, leaflets were stained for 1 hour with ARS, flash frozen in liquid nitrogen and pulverized, followed by 30 minutes incubation with 10% acetic acid at room temperature with shaking. After incubation, samples were heated at 85°C for 10 minutes. Mineral oil was added to the solution to avoid evaporation while heating. Samples were then cooled on ice for 5 minutes and centrifuged at 20000 × g. The supernatant was transferred to a new tube and mixed with 10% ammonium hydroxide. Absorbance of the resulting solution was read at 405 nm on a microplate reader (Synergy HT, Biotek Instruments).

## Results

### Depletion of collagen from the native ECM

**Figure 1:**
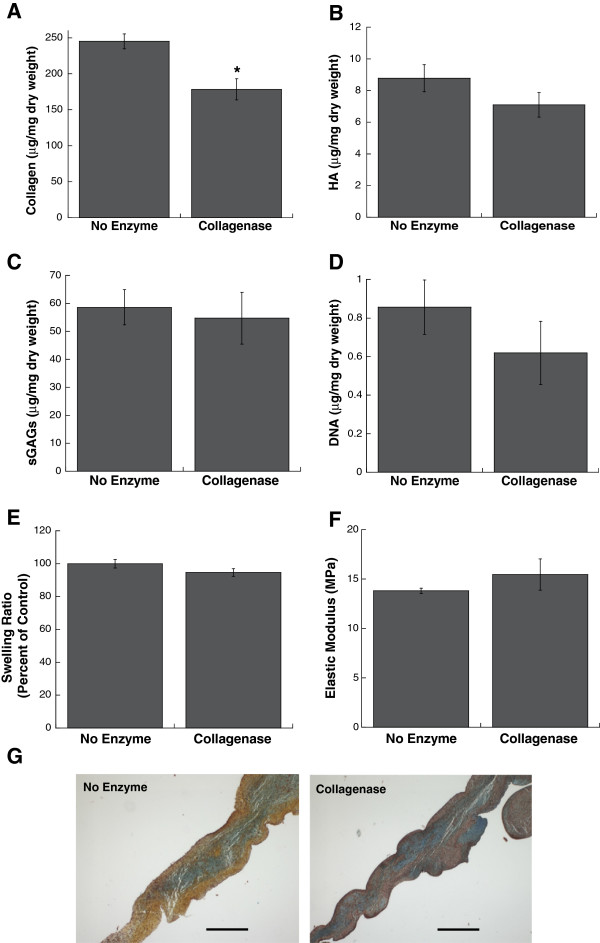
**Leaflet characterization immediately after collagenase treatment, prior to *in vitro *organ culture.** Quantification of **(A)** total collagen, **(B)** HA, **(C)** total sGAGs, **(D)** DNA, **(E)** swelling ratio, and **(F)** Young’s Modulus indicated that collagenase-treated leaflets were selectively depleted of collagen. **(G)** Movat’s Pentachrome staining indicated depletion of collagen (yellow) in collagenase-treated leaflets, but retention of other valve components, such as proteoglycans (blue). Scale bar = 400 μm. *P <0.05 compared to undepleted leaflets (no enzyme).

To better understand the role of collagen in regulating the phenotype and calcification of VICs, we took a “top-down” approach in which collagen was partially removed from native aortic valve leaflets via targeted enzymatic digestion. Although we have previously reported a variation of this technique [5] to explore the contributions of individual ECM components to VIC function in a 3D environment, we first performed validation experiments to quantify collagen loss and retention of other properties. Figure 1 demonstrates that this ECM depletion method is effective in removing solely the target ECM component from the native leaflet. Specifically, collagenase treatment was highly effective in removing 27.2% of the collagen (Figure 1A) without altering other important leaflet characteristics; namely, the amounts of HA (Figure 1B) and sulfated GAGs (Figure 1C), as well as cellularity (Figure 1D), swelling ratio (Figure 1E), and Young’s Modulus (Figure 1F) did not significantly differ between untreated and collagenase-treated leaflets (P > 0.05). Movat’s pentachrome staining also confirmed retention of cellularity and overall leaflet architecture; the proteoglycan-rich spongiosa layer (blue) was not noticeably altered, but diminishment of collagen content (yellow) was obvious upon collagenase treatment (Figure 1G).

### Characterization of collagen-deficient leaflet composition

**Figure 2:**
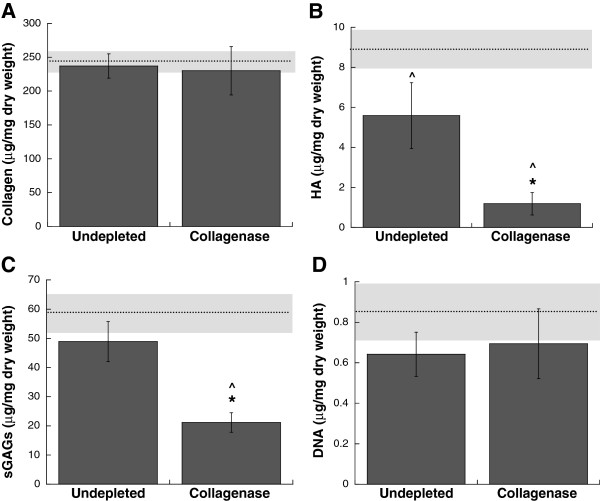
**After 6 days of *in vitro *culture, leaflet composition was analyzed via measurement of (A) total collagen, (B) HA, (C) total sGAGs, and (D) DNA.** The dashed line and gray box in each panel represent the mean and standard deviation for the Day 0 (fresh, undepleted leaflet) control. *P <0.05 compared to undepleted; ^P < 0.05 compared to Day 0 control.

Following confirmation of the feasibility of using a low concentration of collagenase to preferentially decrease leaflet collagen content with minimal disruption of other leaflet ECM components, these altered leaflets were then used to examine how the loss of collagen from the native leaflet environment impacted VIC phenotype. After 6 days of *in vitro* culture, the ECM composition of undepleted leaflets remained similar to the composition of the Day 0 fresh leaflets (Figure 2; Day 0 average is represented as dashed line, with gray box representing Day 0 standard deviation), although HA content did decrease slightly relative to the Day 0 control. Leaflets that had been subjected to collagenase treatment on Day 0 demonstrated an increase in collagen over the subsequent 6 days of culture such that the collagen content of the collagenase-treated condition recovered to levels found in the undepleted Day 0 and Day 6 controls (Figure 2A). Meanwhile, collagenase-treated leaflets displayed a significant decrease in HA and sGAGs after the *in vitro* culture period (Figure 2B-C, P < 0.05). The drop in HA content was particularly notable, resulting in 5-fold less HA than found in the undepleted control. As shown in Figure 2D, collagen disruption did not significantly alter leaflet cellularity from the baseline value obtained for Day 0 fresh leaflets.

### Phenotypic characterization of collagen-deficient leaflets

**Figure 3:**
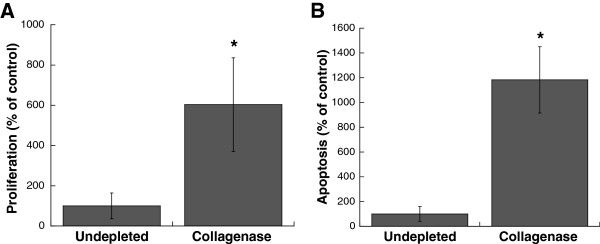
**After 6 days of *in vitro *culture, VICs in collagen-deficient leaflets exhibited significantly higher levels of both proliferation and apoptosis. (A)** Proliferating cells per total cell number on Day 6, and **(B)** Apoptotic cells per total cell number on Day 6, expressed as percent of no-enzyme control. *P <0.0005 compared to undepleted.

**Figure 4:**
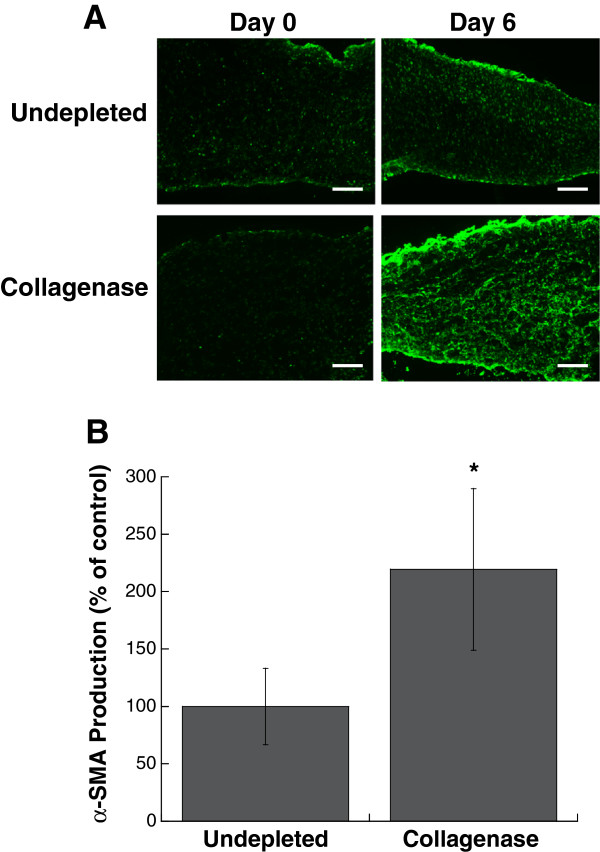
**α-SMA expression by VICs in collagen-deficient leaflets was elevated compared to undepleted leaflets, as evidenced by (A) immunohistochemical staining for α-SMA on Day 6 and (B) western blot for α-SMA on Day 6.** Scale bar = 100 μm. *P < 0.05 compared to undepleted.

**Figure 5:**
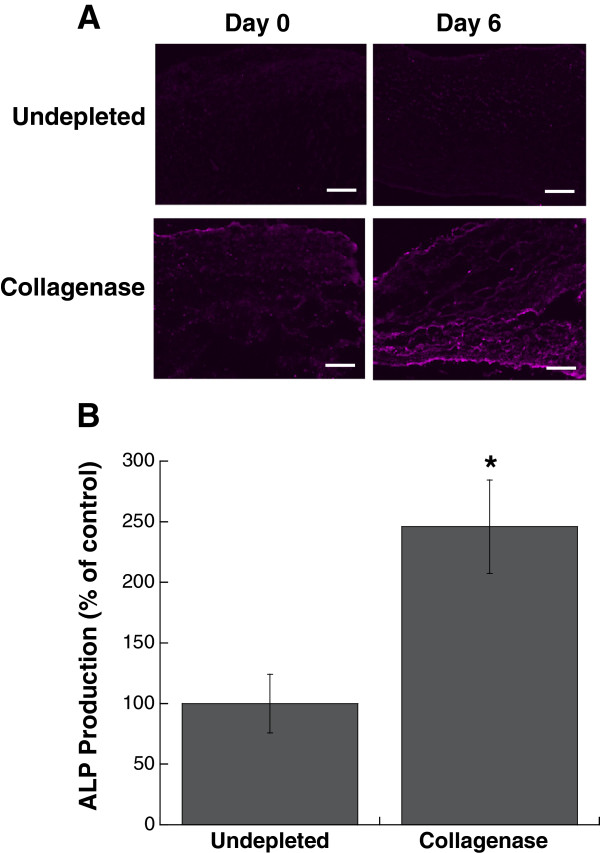
**ALP expression by VICs in collagen-deficient leaflets was elevated compared to undepleted leaflets, as evidenced by (A) immunohistochemical staining for ALP on Day 6 and (B) western blot for ALP on Day 6.** Scale bar = 100 μm. *P < 0.05 compared to undepleted.

**Figure 6:**
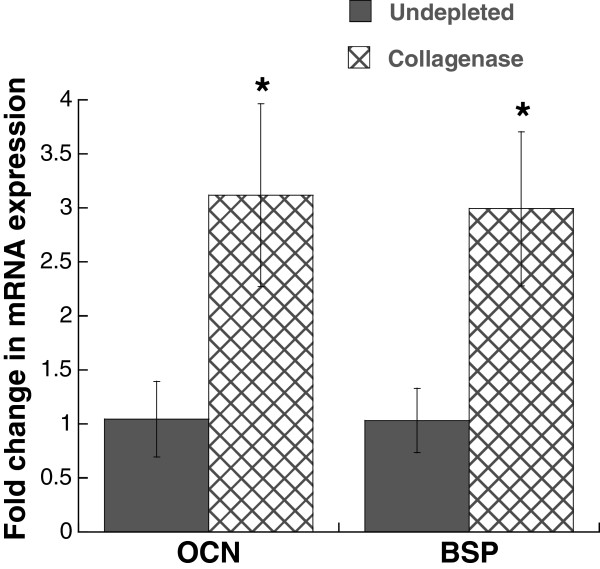
**Expression of later-stage osteoblastic markers osteocalcin (OCN) and bone sialoprotein (BSP) in undepleted vs. collagen-depleted leaflets was measured via RT-PCR after 6 days of *in vitro *culture; both markers were significantly elevated in collagen-deficient leaflets.** *P < 0.05 compared to undepleted.

**Figure 7:**
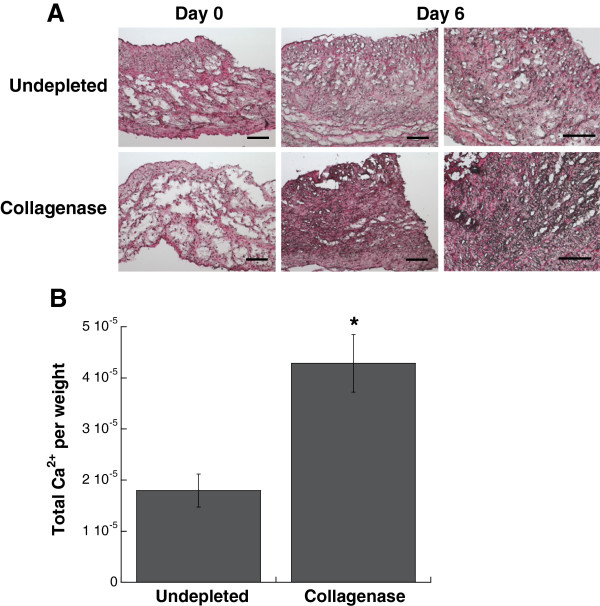
**Characterization of leaflet mineralization indicated greater mineralization of collagen-deficient leaflets compared to undepleted controls. (A)** von Kossa staining for mineralization was performed after 6 days of culture; calcium deposits are stained black. Higher magnification images of Day 6 samples are included to better visualize the differences in staining between conditions. **(B)** Calcium content in collagen-deficient leaflets compared to undepleted leaflets after 6 days of culture. Scale bar = 100 μm. *P < 0.05 compared to undepleted.

Characterization of leaflet cultures demonstrated that the partial removal of collagen from the native leaflet also had a significant impact on VIC proliferation, apoptosis, and phenotype. Collagenase treatment was sufficient to significantly increase both VIC proliferation and apoptosis in leaflets when compared to the undepleted control (Figure 3).Quantification of multiple calcific markers via immunohistochemistry, western blot, and qRT-PCR indicated that the partial removal of collagen resulted in VIC expression of markers associated with myofibroblast and osteoblast activity, both of which are known to contribute to calcification. Specifically, production of α-SMA, the hallmark protein indicative of a myofibroblast VIC phenotype, was significantly increased in collagenase-treated leaflets (Figure 4). ALP, which is used as an early marker for osteogenic differentiation, was also significantly increased in collagen-depleted leaflets (Figure 5). Gene expression analysis indicated that later-stage markers of osteogenesis, OCN and BSP, were also significantly elevated in collagen-depleted leaflet cultures (Figure 6). Finally, these phenotypic changes were found to ultimately result in increased mineralization in collagen-depleted leaflets, as indicated by positive von Kossa staining (Figure 7A) and significantly increased total calcium content (Figure 7B).

## Discussion

Collagen is the most abundant ECM protein in the valve leaflet [14] and has proven attractive as a scaffold material for valve tissue engineering [27,28]. However, relatively little is known about the importance of VIC-collagen interactions in the valve leaflet, particularly with respect to VIC phenotype and calcification. Thus, this study investigated the role of native valve collagen content in regulating VIC phenotype. Our findings suggest that collagen disruption or deficiency is sufficient to induce VIC activation to a pathological phenotype, with a subsequent increase in mineralization.

Calcified valves experience a decrease in collagen content [8], particularly in localized areas surrounding calcific nodules. Meanwhile, work by our own lab has shown that VICs cultured on collagen-coated surfaces did not form nodules, even after treatment with potent pro-calcific stimuli [7], suggesting that collagen may act in a ‘protective’ manner and actively perform functions to inhibit VIC calcification. Thus, to further investigate the importance of VIC-collagen interactions in valve calcification, we manipulated native valve ECM composition via an ECM-depletion method to characterize the phenotypic response of VICs to a decrease in native collagen content. As shown by our results, this method can be tailored to remove from the native environment only the ECM component of interest (in this case, collagen) without significantly altering cellularity, tissue mechanics, or other ECM proteins. Using this method, we were able to successfully remove 27% of the total collagen, thus creating collagen-deficient leaflets. VIC viability within the depleted leaflets also remained at the same level as undepleted controls (not shown), which was the expected result, as the amount of collagenase used for depletion is substantially less than what is typically used to harvest healthy, viable VICs from leaflets for subsequent *in vitro* culture.

The partial removal of collagen was effective at inducing both VIC proliferation and apoptosis relative to undepleted leaflets, which remained relatively quiescent. VIC apoptosis and proliferation are known to be increased in response to injury and to contribute to valve pathology [29-32]. Thus, the increase of these two events upon alteration of ECM content further indicates that VICs were activated in response to these environmental changes.

Previous studies by others suggest that, in calcified valves, changes in the microenvironment, such as collagen damage, may stimulate a series of events that cause accumulation of tenascin-C, an ALP upregulator that participates in mineralization [33]. Our results are consistent with this finding, as depletion of collagen from the valve leaflet led to an increase in the expression of several disease markers, including ALP. Specifically, we observed a significant increase in the protein expression of ALP and in the mRNA expression of OCN and BSP, all markers of an osteoblast-like phenotype. Production of α-SMA, indicative of a myofibroblast phenotype, was also upregulated in the collagen-deficient leaflets. Our mineralization data further demonstrate that interaction of VICs with collagen is important for the prevention of a diseased state, as disruption of collagen homeostasis was sufficient to increase calcium content in leaflet cultures. Together, these results suggest that inducing a collagen-deficient leaflet environment led to the stimulation of VICs to differentiate into pathological phenotypes, ultimately resulting in initiation of calcification.

It is interesting to note that the initial disruption in collagen resulted in a dramatic decrease in leaflet HA content, considering that previous work by our group has identified VIC-HA interactions as being crucial for maintenance of a healthy VIC phenotype [5]. In fact, the elevation of proliferation, apoptosis, ALP, and α-SMA described herein mirrors findings reported for when VIC-HA interactions were disrupted via a blocking antibody or when leaflets were rendered HA-deficient [5]. The authors have not found this to occur when other ECM components, such as elastin or chondroitin sulfate, are depleted (via elastase or chondroitinase, respectively; see Additional file 1: Figure S1), so this does not appear to merely be a non-specific result related to leaflet ECM alteration. These similarities, combined with the fact that collagen depletion induced an HA deficiency in the current study, raises the question of whether the phenotype results described herein are actually due to disruptions in VIC-HA interactions, with collagen insufficiency being the event that initiates this cascade of events. The ability of collagen disruptions to act as a precipitating event to HA disruption and subsequent phenotypic alterations and calcification cannot be confirmed by the current study, but forms an interesting question to investigate in future work to better understand ECM dynamics in valve dysfunction.

Finally, it is important to note one significant limitation in this study, which is that the experiments were performed under static, rather than dynamic, conditions. Although we were generally able to retain the structural and phenotypic characteristics of undepleted valves during the 6-day static culture, several studies have demonstrated differences in cellular outcomes when comparing static to dynamic leaflet culture [34-36]. Thus, it is possible that this work would yield different results upon exposure of the leaflets to dynamic mechanical stimulation. There exist many types of valve-specific bioreactors that differ widely in their configurations and abilities (*e.g.*, flow, stretch, direction of stretch, uniaxial vs. biaxial, flexure, etc.) [34-39], but one significant caveat associated with their use is a lack of clarity regarding what sort of dynamic conditioning renders the most meaningful results (*i.e.*, it is not known whether any type of mechanical conditioning will always be more physiologically relevant than none at all).

## Conclusions

Overall, the findings presented herein emphasize the importance of collagen-rich environments in maintaining a healthy, quiescent VIC phenotype and indicate that a deficiency in endogenous collagen content is sufficient to lead to a diseased VIC phenotype and subsequent leaflet mineralization. By successfully removing collagen from the native ECM without impacting cellularity and tissue structure, this work may also contribute to the goal of creating “synthetically-diseased” leaflets that can be used as *in vitro* models to study valvular disease progression and target possible treatments. Characterizing the importance of collagen in valve function may help in the design of scaffolds for valve tissue engineering, as well as contribute to our understanding of the events involved in CAVD.

## Competing interests

The authors have no competing interests to declare.

## Authors’ contributions

KR and LP participated in leaflet characterization and phenotype analyses. AP participated in leaflet characterization. KR, LP, and AP participated in the design of the study, performed statistical analyses, and participated in data interpretation. KM conceived of the study and participated in its design and coordination and in data interpretation. KR and KM drafted the manuscript. All authors read and approved the final manuscript.

## Pre-publication history

The pre-publication history for this paper can be accessed here:

http://www.biomedcentral.com/1471-2261/14/29/prepub

## Supplementary Material

Additional file 1: Figure S1 Leaflets were depleted of elastin using 20 U/mL elastase following procedures similar to those outlined for collagen depletion. After 6 days of culture, elastin-depleted leaflets were histologically analyzed for: **(A)** mineralization via von Kossa staining, **(B)** α-SMA expression, and **(C)** ALP expression. Detectable mineralization, α-SMA, and ALP were not found in any of the elastin-depleted leaflets. Scale bar = 100 μm. Click here for file
