# Supplementary material for: Manipulation of valve composition to elucidate the role of collagen in aortic valve calcification
Source: BMC Cardiovasc Disord. 2014 Mar 1;14:29. doi: 10.1186/1471-2261-14-29 (PMC3946110; doi:10.1186/1471-2261-14-29)
Supplement: Additional file 1: Figure S1 — Leaflets were depleted of elastin using 20 U/mL elastase following procedures similar to those outlined for collagen depletion. After 6 days of culture, elastin-depleted leaflets were histologically analyzed for: (A) mineralization via von Kossa staining, (B) α-SMA expression, and (C) ALP expression. Detectable mineralization, α-SMA, and ALP were not found in any of the elastin-depleted leaflets. Scale bar = 100 μm. [file 1471-2261-14-29-S1.pdf]

DATA SUPPLEMENT

A

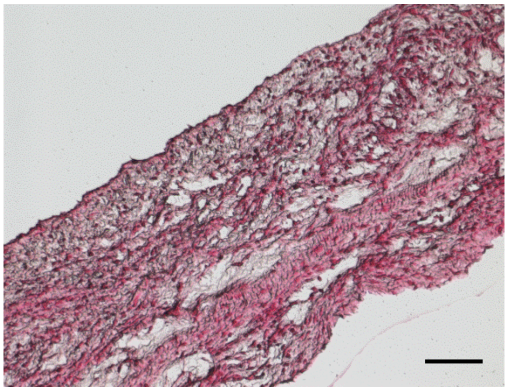

B

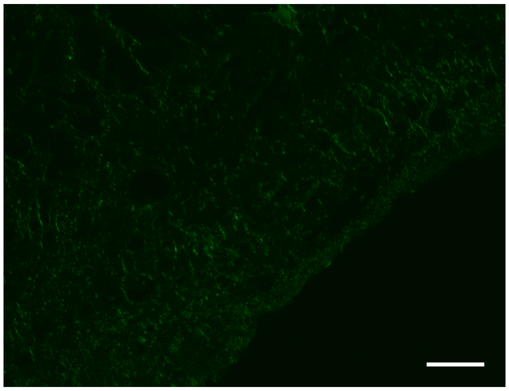

C

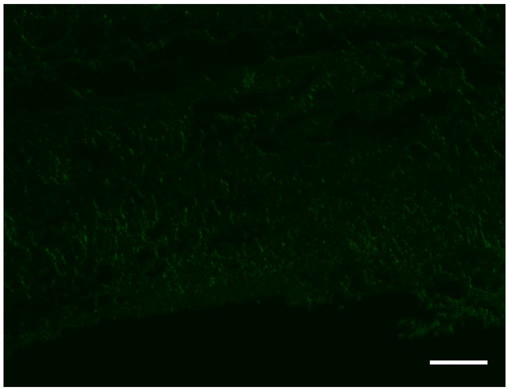

Supplementary Figure S1. Leaflets were depleted of elastin using 20 U/mL elastase following procedures similar to those outlined for collagen depletion. After 6 days of culture, elastin-depleted leaflets were histologically analyzed for: (A) mineralization via von Kossa staining, (B) α-SMA expression, and (C) ALP expression. Detectable mineralization, α-SMA, and ALP were not found in any of the elastin-depleted leaflets. Scale bar = 100 μm.
